# Supplementary material for: Pilates to Improve Core Muscle Activation in Chronic Low Back Pain: A Systematic Review
Source: Healthcare (Basel). 2023 May 12;11(10):1404. doi: 10.3390/healthcare11101404 (PMC10218154; doi:10.3390/healthcare11101404)
Supplement: Supplementary file 1 [file healthcare-11-01404-s001.zip › healthcare-2344428-Supplementary final-revise.pdf]

Supplementary Table S1. OVID (Medline) database search strategy.

Search ID#	Search Terms	Search Notes
S1	exp Back Pain/	P concept – MeSH.
S2	"back pain".mp. [mp=title, book title, abstract, original title, name of substance word, subject heading word, floating sub-heading word, keyword heading word, organism supplementary concept word, protocol supplementary concept word, rare disease supplementary concept word, unique identifier, synonyms]	P concept – keyword (m.p 'multi-purpose' searching all of the fields listed)
S3	("low* back" adj3 pain).mp	P concept – keyword
S4	("back ache" or backache or "back-ache").mp	P concept – keyword
S5	(back adj3 disorder*).mp	P concept - keyword
S6	(lumbar adj3 pain).mp.	P concept - keyword
S7	exp Spondylosis/	P concept – MeSH.
S8	Spondylosis.mp.	P concept - keyword
S9	Intervertebral Disc Degeneration/	P concept – MeSH.
S10	Intervertebral Disc Displacement/	P concept – MeSH.
S11	(disc adj3 (degenerat* or prolaps* or hernia*)).mp.	P concept - keyword
S12	Lumbosacral Region/	P concept – MeSH.
S13	((lumbar or lumbo*) adj3 region*).mp.	P concept - keyword
S14	exp Spine/	P concept – MeSH.
S15	spine.mp.	P concept - keyword
S16	1 or 2 or 3 or 4 or 5 or 6 or 7 or 8 or 9 or 10 or 11 or 12 or 13 or 14 or 15	Combined searches for P concept
S17	Exercise Movement Techniques/	I concept – MeSH<br>NB: Scope note says '…use for Pilates…'

S18	Pilates.mp.	I concept - keyword
S19	17 or 18	Combined searches for I concept
S20	16 and 19	Combined P and I concepts

Supplementary Table S2. Reasons for exclusion at full text review.

Reference	Reason for exclusion
MacHado, P. M.; Alves, M. C.; Hendler, K. G.; Benetti, V. B.; De Souza Neto, R. J.; Barbosa, R. I.; Marcolino, A. M.; Kuriki, H. U. Motriz 2018;Revista de Educacao Fisica. 23(4) (no pagination)():2018 DOI: 10.1590/S1980-6574201700040009	Wrong study design
Alves, M. C.; de Souza Neto, R. J.; Barbosa, R. I.; Marcolino, A. M.; Kuriki, H. U.Clinical Biomechanics February 2020;72():172-178 2020 February DOI: 10.1016/j.clinbiomech.2019.12.009	Wrong outcomes
Palanichamy, S.; Rajeswari, International Journal of Pharmaceutical Research April-June 2021;13(2)():1726-1732 2021 April-June DOI: 10.31838/ijpr/2021.13.02.209	Wrong outcomes
Torres Cruz, A.; De Oliveira Januario, P.; Coelho Baptista, I.; Da Rocha Rodrigues, A.; Libanio De Souza Castro, B.; Bezerra Oliveira, G.; Da Silva Palmeira, I.; De Fatima Cabral Dos Santos, T.; Pasqual Marques, A.Annals of the Rheumatic Diseases June 2022;81(Supplement 1)():1870-1871 2022 June DOI: 10.1136/annrheumdis-2022-eular.1388	Conference abstract
Morkved, S.; Salvesen, K. A.; Schei, B.; Lydersen, S.; Bo, K. Acta Obstetricia et Gynecologica Scandinavica 2007;86(3):276-82 2007	Wrong intervention
Oksuz, S.; Unal, E.; Arin, G. Annals of the Rheumatic Diseases June 2018;77(Supplement 2)():1852 2018 June DOI: 10.1136/annrheumdis-2018-eular.3980	Conference abstract
Hides, J. A.; Stanton, W. R.; Mendis, M. D.; Gildea, J.; Sexton, M. J. Medicine and Science in Sports and Exercise June 2012;44(6)():1141-1149 2012 June DOI: 10.1249/MSS.0b013e318244a321	Wrong participants
Baskan, O.; Cavlak, U.; Baskan, E. Annals of Clinical and Analytical Medicine 2021;12(Supplement 4)():478-482 2021 DOI: 10.4328/ACAM.20648	Wrong outcomes
Baglan Yentur, S.; Sarac, D. C.; Sar, I. F.; Tore, N. G.; Satis, H.; Ozturk, M. A.; Oskay, D. Annals of the Rheumatic Diseases June 2020;79(SUPPL 1)():912 2020 June DOI: 10.1136/annrheumdis-2020-eular.2129	Conference abstract
Torres Cruz, A.; De Oliveira Januario, P.; Coelho Baptista, I.; Da Rocha Rodrigues, A.; Chagas Bernardo, C. H.; Silva Nunes, T.; Antunes, M.; Merllin Batista De Souza, I.; Pasqual Marques, A. Annals of the Rheumatic Diseases June 2020;79(SUPPL 1)():1949 2020 June DOI: 10.1136/annrheumdis-2020-eular.3371	Conference abstract
Rydeard, R.; Leger, A.; Smith, D. Journal of Orthopaedic & Sports Physical Therapy 2006;36(7):472-484 Alexandria, Virginia JOSPT, Inc. d/b/a Movement Science Media 2006	Wrong outcomes

Mayana, K. Isah; Ahmad, A. Khamis Physiotherapy 2020;107():e162-e162 Philadelphia, Pennsylvania Elsevier B.V. 2020 DOI: 10.1016/j.physio.2020.03.237	Conference abstract
Herrington, L.; Davies, R. Journal of Bodywork and Movement Therapies January 2005;9(1)():52-57 2005 January DOI: 10.1016/j.jbmt.2003.12.005	Wrong participants
Kofotolis, N.; Kellis, E.; Vlachopoulos, S. P.; Gouitas, I.; Theodorakis, Y. Journal of Back & Musculoskeletal Rehabilitation 2016;29(4):649-659 2016	Wrong outcomes
de Barros Silveira, Aline Prieto; Nagel, Laura Zanforlin; Dias Pereira, Dayane; Kazue Morita, Angela; Hebling Spinoso, Deborah; Tavella Navega, Marcelo; Ribeiro Marques, Nise ConScientiae Saude 2016;15(2):231-240 Nove de Julho University 2016 DOI: 10.5585/ConsSaude.v15n2.6407	Non-English language
Kanase, S. B.; Patil, S. K. Journal of Ecophysiology and Occupational Health December 2021;21(4)():164-167 2021 December DOI: 10.18311/JEOH/2021/28914	Wrong study design
de Barros Silveira, Aline Prieto; Nagel, Laura Zanforlin; Dias Pereira, Dayane; Morita, Ângela Kazue; Spinoso, Deborah Hebling; Navega, Marcelo Tavella; Marques, Nise Ribeiro Fisioterapia e Pesquisa 2018;25(2):173-181 Universidade de Sao Paulo, Faculdade de Medicina 2018 DOI: 10.1590/1809-2950/17594425022018	Wrong study design
Schroeder, Jan pt Zeitschrift für Physiotherapeuten 2011;63(11):6-12 Richard Pflaum Verlag GmbH & Co. KG 2011	Wrong study design
Marshall, P. W. M.; Kennedy, S.; Brooks, C.; Lonsdale, C. Spine 01 Jul 2013;38(15)():E952-E959 2013 01 Jul DOI: 10.1097/BRS.0b013e318297c1e5	Wrong outcomes
Castro, J. B.; Lima, V. P.; Mello, D. B.; Lopes, G. C.; Peixoto, J. C.; Santos, A. O. D.; Nunes, R. A.; Souza Vale, R. G. Pain Management 2022;12(4):509-520 2022	Wrong outcomes
Kawanishi, Carolinne Y.; de Oliveira, Márcio R.; Coelho, Vinícius S.; Parreira, Rodolfo B.; de Oliveira, Rodrigo F.; Santos, Cléssius F.; da Silva, Rubens A. Revista Terapia Manual 2011;9(44):410-417 Revista Terapia Manual 2011	Non-English language
Emery, K.; De Serres, S. J.; McMillan, A.; Cote, J. N. Clinical Biomechanics February 2010;25(2)():124-130 2010 February DOI: 10.1016/j.clinbiomech.2009.10.003	Wrong participants
Coelho, V. S.; Kozu, A. H.; dos Santos, C. F.; Vitor, L. G. V.; Gil, A. W.; de Oliveira, M. R.; Parreira, R. B.; da Silva, R. A. Revista Terapia Manual 2010;8(40):508-516 Revista Terapia Manual 2010	Non-English language
Mazloum, V.; Sahebozamani, M.; Barati, A.; Nakhaee, N. Journal of Mazandaran University of Medical Sciences December 2016;26(143)():48-61 2016 December	Non-English language
Montero-Cámara, J.; Sierra-Silvestre, E.; Monteagudo-Saiz, A. M.; López-Fernández, J.; López-López, A. l; Barco-Pérez, M. E. Fisioterapia 2013;35(5):206-213 New York, New York Elsevier B.V. 2013 DOI: 10.1016/j.ft.2012.10.004	Wrong participants
Wawryszewicz, M.; Targosinski, P. Advances in Rehabilitation 2020;34(3)():22-28 2020 DOI: 10.5114/areh.2020.99031	Wrong participants
[22] Mazloum, Vahid; Sahebozamani, Mansour; Barati, Amirhossein; Nakhaee, Nouzar; Rabiei, Pouya Journal of Bodywork & Movement Therapies 2018;22(4):999-1003 Churchill Livingstone, Inc. 2018 DOI: 10.1016/j.jbmt.2017.09.012	Wrong outcomes
Haiou, N.; Jinyu, Y.; Ye, D.; Yan, W.; Zhiyu, J.; Jianfeng, L.; Yong, Z. Physiotherapy (United Kingdom) May 2015;1)():eS504 2015 May DOI: 10.1016/j.physio.2015.03.3304	Wrong study design

Kistler-Fischbacher, M.; Yong, J. S.; Weeks, B. K.; Beck, B. R. Journal of Bone and Mineral Research September 2021;36(9)():1680-1693 2021 September DOI: 10.1002/jbmr.4334	Wrong participants
Barbosa Silva, Daiane Karine; Murata, Eduardo; de Freitas, Cíntia Domingues Revista Terapia Manual 2013;11(51):90-94 Revista Terapia Manual 2013	Non-English language
Borges, V.; Junqueira, C.; Moreira, S. S.; Ferreira, J.; Drumond Mitre, N. C.; Tamara da Silva Lage, P.; Carvalho Mitre Chaves, C. M. Archives of Physical Medicine and Rehabilitation December 2019;100(12)():e176 2019 December DOI: 10.1016/j.apmr.2019.10.042	Conference abstract
Ferri-Caruana, A.; Romagnoli, M.; Salazar-Bonet, L. R.; Staiano, W. medRxiv. 2022;10(): 2022 DOI: 10.1101/2022.03.07.22270395	Wrong outcomes
Kliziene, I.; Sipaviciene, S.; Vilkiene, J.; Astrauskiene, A.; Cibulskas, G.; Klizas, S.; Cizauskas, G. Journal of Bodywork and Movement Therapies 01 Jan 2017;21(1)():124-132 2017 01 Jan DOI: 10.1016/j.jbmt.2016.06.005	Wrong outcomes
Sakabe, Daniel Iwai; Sakabe, Fabiana Forti; Dias de Souza, Ariane Cristina; Guerreiro, Alline Pereira Manual Therapy, Posturology & Rehabilitation Journal 2020;18():1-6 Revista Terapia Manual 2020 DOI: 10.17784/mtprehabjournal.2020.18.790	Wrong study design
Luk, T. H.; Critchley, D. J. Physiotherapy (United Kingdom) June 2011;1)():eS718-eS719 2011 June DOI: 10.1016/j.physio.2011.04.002	Conference abstract
O'Brien, N.; Hanlon, M.; Meldrum, D. Physical Therapy Reviews 2006;11(3):224-225 Philadelphia, Pennsylvania Taylor & Francis Ltd 2006	Conference abstract
Kutlu Ozkaraoglu, D.; Dinc Yavas, A.; Akgun, A. S.; Algun, Z. C. Osteoporosis International December 2020;31(SUPPL 1)():S562 2020 December DOI: 10.1007/s00198-020-05696-3	Conference abstract
Kim, Y.; Son, J.; Yoon, B. European Journal of Applied Physiology 2013;113(4):997-1004 2013	Wrong study design
Andrade, L. S.; Mochizuki, L.; Pires, F. O.; da Silva, R. A.; Mota, Y. L. Journal of Bodywork & Movement Therapies 2015;19(1):62-6 2015	Wrong study design
Sharma, Swati; Sarin, Avnee Indian Journal of Physiotherapy & Occupational Therapy 2017;11(4):98-104 Institute of Medico-legal publications Pvt Ltd 2017 DOI: 10.5958/0973-5674.2017.00128.9	Wrong study design

Supplementary Table S3. Study eligibility criteria and participant retention.

Study	Eligibility Criteria	Recruitment		Retention	
		Number (%)	Reasons	Number (%)	Reasons
Ashtiani (2020) [58]	Chronic low back pain, Irritation/stiffness in LSP region with/without lower limb referral (without root cause), >3 month history, 18-50 years.<br>No history of vertebral fracture, pregnancy, tumour, infection , previous spinal surgery, serious spinal deformity, cardiovascular disorder, central nervous system disorder, vestibular disorder, visual disorder neurological symptoms including sensory defects or motor paralysis, no neurologic or rheumatic disease.	30 (100%)		Exp: 15/15 (100%)<br>Con: 15/15 (100%)	
Batibay (2020) [59]	CNSLBP for > 3 months, VAS score 3-6/10; females aged 18-60 years.	60 (100%)		Exp: 28/30 (93%)<br>Con: 25/30 (83%)	Exp: insufficient attendance due to work commitments<br>Con: unable to follow exercise programmes
Bhadauria (2017) [56]	M/F adults aged 20-60 years; history of CNSLBP >3 months; subjects willing to participate in study	52 screened 44 recruited (85%)	8 excluded:<br>4 did not meet inclusion criteria<br>2 refused treatment<br>2 cited other reasons	MP: 12/15 (80%)<br>LS: 12/15 (80%)<br>DS: 12/14 (86%)	MP: 2 family problem; 1 personal reason<br>LS: 2 missed visit; 1 fell<br>DS: 2 health problem
Brooks (2012) [57]	M/F adults aged 18-50 years; history of CNSLBP >12 weeks	132 screened 64 recruited (48%)	68 excluded:<br>62 did not meet inclusion criteria<br>5 declined to participate<br>1 cited other reasons	Exp:29/32 (91%)	Exp: 1 family illness; I injury at work; 1 dissatisfied with intervention

				Con: 23/32 (72%)	Con:<br>2 lost to follow up (1 time; 1 family illness)<br>7 discontinued intervention (1 attendance difficulty; 1 illness; 5 dissatisfied with intervention)
Cruz-Diaz (2017) [60]	Participants aged between 18-50 years; pain between 3-7/10 on VAS; history of >12 weeks LBP pain; absence of radiculopathy or other damage to spine such as fractures, tumours or stenosis; not habitual Pilates practitioners; not receiving other physiotherapy treatment during or immediately prior to trial (previous 6 months); not pregnant; enough physical autonomy to participate in the physical activities required of the study	132 screened<br>102 recruited (77%)	30 excluded:<br>26 did not meet inclusion criteria<br>4 declined to participate	MP: 34/34 (100%)<br>AP: 34/34 (100%)<br>Con: 30/34 (88%)	Con: 4 lost for inability to contact
Mendes Tozim (2021) [61]	Presence of LBP or exacerbation of chronic LBP for at least 6 months prior to study	66 screened<br>46 recruited (70%)	20 excluded:<br>20 did not meet inclusion criteria	MP; 14/16 ((88%)<br>GE: 13/15 (87%)<br>EG: 14/15 (93%)	MP: 2 no time<br>GE: 1 no time; 1 disease<br>EG: 1 no time
Nabavi (2017) [62]	Good general health (using Farsi version of 12-item General Health Q/A); suffering from CNSLBP (with no clear pathological cause) for >12 weeks.	55 screened<br>41 recruited (75%)	14 excluded:<br>10 did not meet inclusion criteria<br>4 declined to participate	Exp: 20/20 (100%)<br>Con: 21/21 (100%)	
Sonmezer (2021) [63]	Pregnant women at 22-24 weeks gestation with pregnancy induced LBP; maternal age 20-35; parity ≤3; absence of pre-pregnancy lumbar pain	58 screened<br>50 recruited (86%)	8 excluded:<br>4 did not meet inclusion criteria<br>4 declined to participate	Exp: 20/26 (77%)<br>Con: 20/24 (83%)	Exp: 6 lack of motivation<br>Con: 4 social reasons

AP: apparatus pilates; Con: control; CNSLBP: Chronic non specific low back pain; DS: dynamic strengthening; EG: educational group; Exp: experimental; F: female; GE: general exercise group; LBP: low back pain; LSP: lumbar spine; LS: lumbar strengthening; M: male; MP: mat pilates; NR: not recorded; Q/A: questionnaire; sEMG: surface EMG; VAS: visual analogue scale;
